# Tender Nasal Traumatic (TNT) Neuroma: Case Report and Review

**DOI:** 10.7759/cureus.30957

**Published:** 2022-11-01

**Authors:** Philip R Cohen, Antoanella Calame

**Affiliations:** 1 Dermatology, University of California, Davis Medical Center, Sacramento, USA; 2 Dermatology/Dermatopathology, Compass Dermatopathology, San Diego, USA; 3 Dermatology, Scripps Memorial Hospital, La Jolla, USA

**Keywords:** tumor, traumatic, tender, skin, painful, neuroma, neoplasm, nasal, dermal, cutaneous

## Abstract

A traumatic neuroma occurs at the injury site of a peripheral nerve; however, albeit rarely, this variant of a neuroma can involve a nerve that has not experienced penetrating trauma. A lower extremity amputation stump is the most common location of a traumatic neuroma. Traumatic neuromas may be symptomatic; tumor-associated pain can be severe and significantly affect the patient’s quality of life. Several hypotheses have been postulated for the pathogenesis of neuroma-related pain, including alpha-smooth muscle actin, neural fiber structural changes, nerve growth factor, and/or sensitization of the affected nerve. In addition to prevention, non-surgical treatment (such as chemical interventions, cryotherapy, neuromodulation, pharmacologic agents, and physiotherapy) and surgical interventions (such as direct nerve repair at the time of injury or ligation of the nerve proximal to the neuroma and various potential methods to minimize subsequent irritation of the distal free end of the proximal nerve) have been used to manage neuroma-associated pain. A traumatic neuroma of the nose is rare. Indeed, it has only been described in three individuals: two women (including the Caucasian woman in this report and a Turkish woman) and one man. The benign tumor was extremely painful in both women; however, the man’s lesion was non-tender. Prior trauma to the nasal site included either a laceration or elective surgery; however, the reported woman did not experience any penetrating trauma to her nose. The diagnosis was established following an excisional (for the man), incisional (for the Turkish woman), or punch (for the Caucasian woman) biopsy. Follow-up was provided for two of the patients. The man’s neuroma had been completely excised, and he never developed tumor-associated tenderness. However, the pain persisted after the biopsy healed for the reported woman whose neuroma was not entirely removed. The explosive and markedly severe character of the reported patient’s lesion-related tenderness prompted us to propose an acronym for this uncommon yet exquisitely painful variant of a neuroma: tender nasal traumatic (TNT) neuroma.

## Introduction

A neuroma is a benign lesion. It is composed of nerve tissue. The nasal mucosa can be the site of neuromas in patients with multiple endocrine neoplasia 2B (MEN2B) [1-3]. 

A traumatic neuroma is a variant of the tumor that usually develops following injury to the nerve [4-9]. They are frequently located at an amputation site, or on an acral area of an extremity (such as the digit of the hand), or on the plantar surface of the toes (such as a Morton’s neuroma) [10-13]. The nose is a rare site for a traumatic neuroma [14,15].

The characteristics of a woman with a tender nasal traumatic (TNT) neuroma are described. Also, the features of other patients in whom a traumatic neuroma of the nose has been reported are summarized. In addition, the approaches to the management of an individual with a painful traumatic neuroma are reviewed.

## Case presentation

A 64-year-old Caucasian woman presented with severe tenderness of her nose. She had rosacea, affecting her cheeks and nose, of several years duration. Her nose had been painful--particularly when touched--for approximately three years; the symptoms began as a tender spot on the nasal tip, had progressed to involve her entire nose and currently felt as though there were needles inside her nose that were sticking her. In addition, she was especially uncomfortable when the facial mask she was wearing, as a precautionary measure against coronavirus disease 2019 (COVID-19), would contact her nose. However, at times the severe tenderness would suddenly erupt in an explosive manner without any precipitating factor.

She had previously been evaluated by her primary care physician, dermatologists, and otolaryngologists without establishing a diagnosis for her nose pain. She had no history of prior surgery or penetrating trauma to her nose; however, she mentioned that recurrent episodes of redness and soreness--possibly caused by insect bites, inflammation of the hair follicles, or flares of her rosacea--had occurred. Daily treatment with oral doxycycline for one month provided no relief of her symptoms. The medications she took to relieve the pain resulting from some of her medical conditions did not improve her nasal symptoms.

Her medical history was significant for diabetes mellitus, fibromyalgia, hyperlipidemia, hypertension, hypothyroidism, and lower back pain. Her daily oral medications included aspirin, atenolol, atorvastatin, gabapentin, metformin. In addition, she would take naproxen or tramadol when the pain from her fibromyalgia or lower back became more severe.

**Figure 1:**
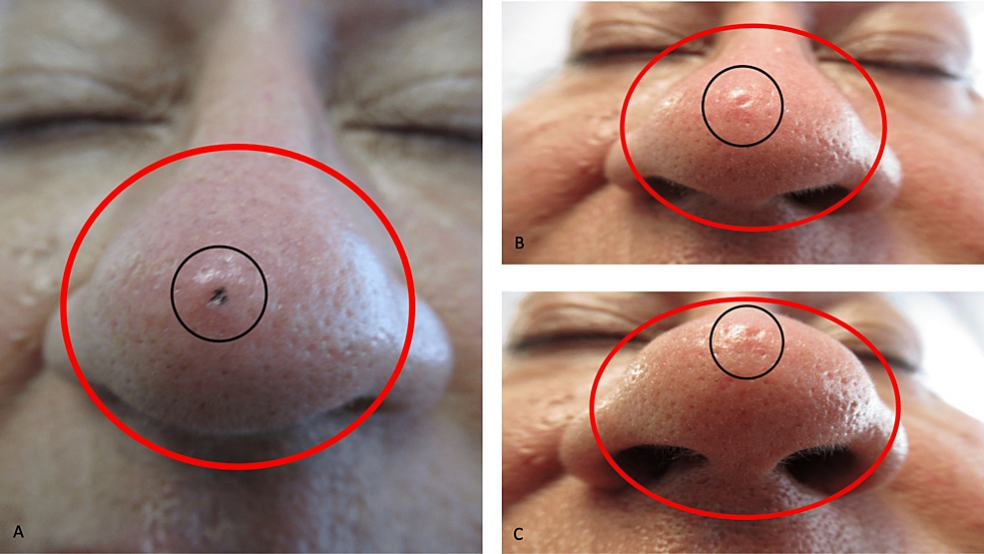
Morphologic features of a TNT (tender nasal traumatic) neuroma Closer frontal (A), distant frontal (B), and distant inferior (C) views of a painful neuroma on the nose of a 64-year-old Caucasian woman of three years duration. Both cheeks are red (B and C), and the bulbous tip of her nose (within the red oval) is erythematous (A, B, and C). A prominent four-by-four millimeters papule with an umbilicated central depression (within the black circle) is also noted (A, B, and C). The purple dot (A) shows the location of the three-millimeter punch biopsy that was performed.

Cutaneous examination of her face showed malar erythema. Her nose was large with an erythematous bulbous tip without rhinophymatous changes; there was a prominent papule, which measured four-by-four millimeters, with a central depression at the nasal tip (Figure 1). A three-millimeter biopsy from the tip of her nose, which included the depressed area of the papule, was performed using the punch technique.

**Figure 2:**
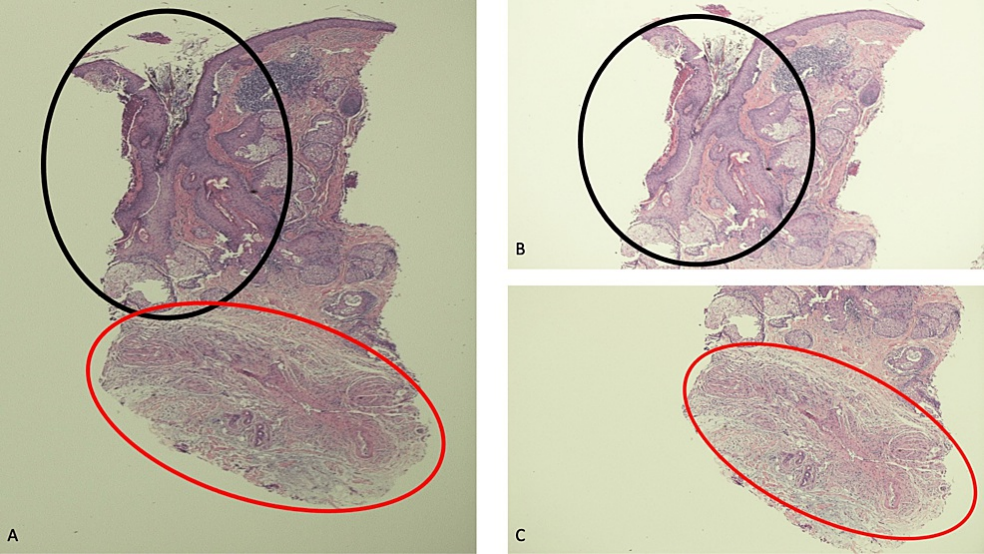
Pathologic features of a TNT neuroma on the nose of a 64-year-old Caucasian woman The upper portion of the biopsy specimen from the nasal tip (within the black oval) showed sebaceous glands adjacent to a structure that extended from the surface of the epithelium into the underlying dermis; it contained a widely dilated follicular opening and a sinus-like epithelial tract lined by the acanthotic epidermis (A and B). In the deeper dermis (within the red oval), there was a tumor consisting of nerve fibers presenting as a proliferation of bundles of spindle-shaped cells (A and C) [Hematoxylin and eosin: A, x2; B, x2; C, x2].

Microscopic evaluation of the tissue specimen, after staining with hematoxylin and eosin, showed a patulous follicular opening in the upper portion of the specimen. The follicle structure contained a widely dilated sinus-like epithelial tract lined by acanthotic epidermis. Adjacent to the follicle, there were numerous prominent sebaceous glands (Figure 2).

**Figure 3:**
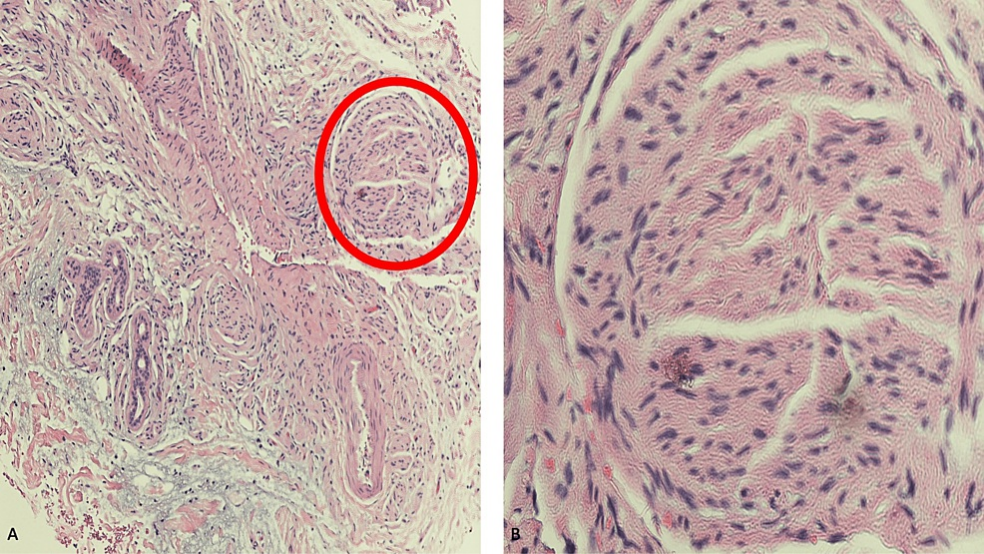
Microscopic changes of a traumatic nasal neuroma Higher magnification views (A and B) of the pathologic features of the painful neuroma on the woman’s nasal tip showed a benign tumor consisting of an irregular interlacing pattern of nerve fiber bundles that are present in the fibrous dermal stroma. Microscopic features of one of the bundles of nerve fibers (within the red oval) are highlighted on the distant (A) and closer (B) views of her tissue specimen [Hematoxylin and eosin: A, x10; B, x40].

In the deeper dermis, there was a proliferation of bundles of spindle-shaped cells. The cells were characteristic of nerve fibers. They appeared as bundles with an irregular interlacing pattern and were present in the fibrous dermal stroma (Figures 2, 3). 

**Figure 4:**
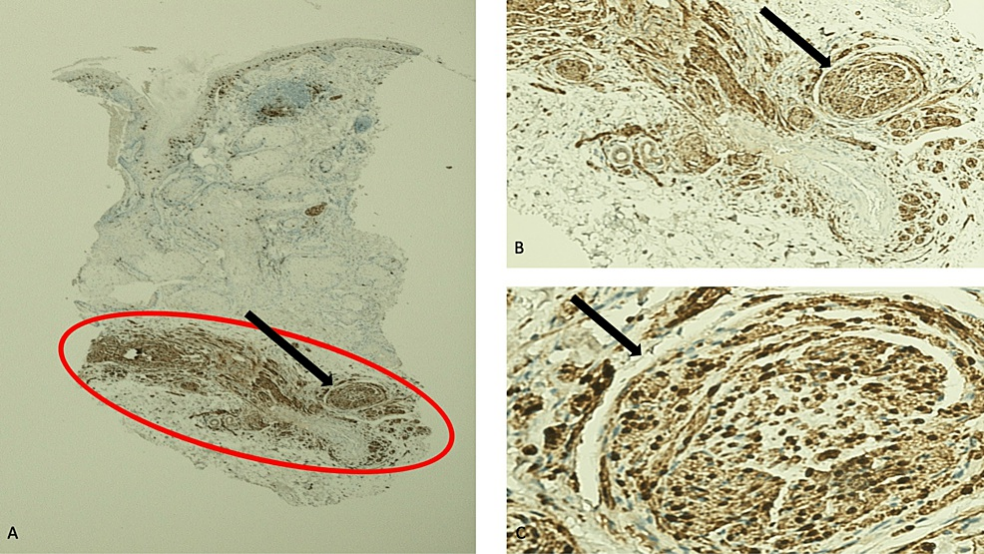
Solubility of proteins in 100 percent ammonium sulfate (S100) staining of a TNT neuroma Lower (A) and higher (B and C) magnification views of the pathologic features of the nasal neuroma (within the red oval) demonstrated that all the spindle-shaped tumor cells present in the reticular dermis showed strongly positive diffuse staining with S100 immunoperoxidase stain. The black arrow highlights one of the bundles of nerves in the benign neural tumor [S100 immunoperoxidase stain: A, x2; B, x10; C, x40].

**Figure 5:**
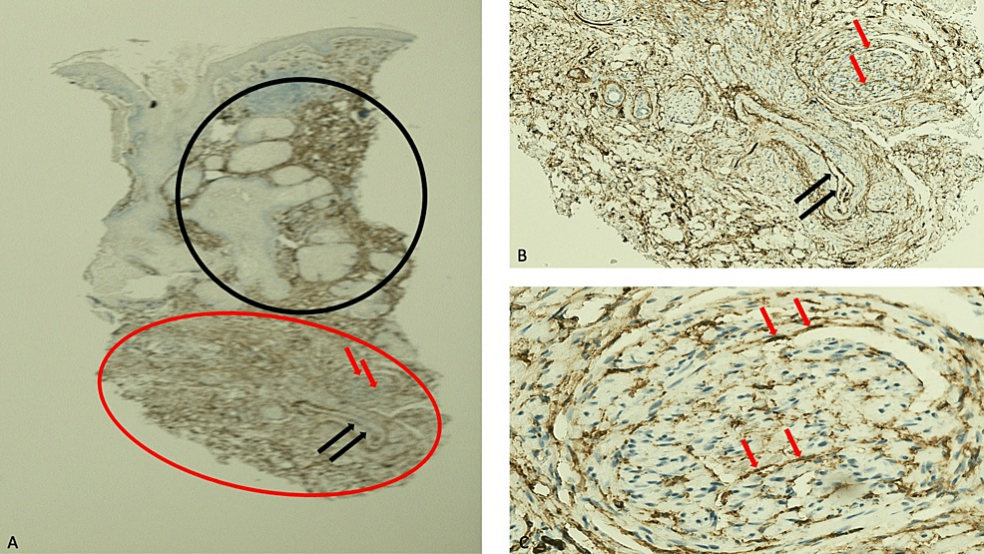
A cluster of differentiation 34 (CD34) staining of a traumatic neuroma on the nose Lower (A) and higher (B and C) magnification views of the microscopic changes of the nasal neuroma in the reticular dermis (within the red oval) demonstrated moderately positive diffuse staining with CD34 immunoperoxidase stain of the neural tumor (red arrows) and faint to weakly positive diffuse staining of the dermal stroma. Positive staining with CD34 immunoperoxidase stain was also observed in some of the normal components of the epithelium in the upper dermis (within the black oval) and in the endothelial cells of vessels in the deeper dermis (black arrows) [CD34 immunoperoxidase stain: A, x2; B, x10; C, x40].

**Figure 6:**
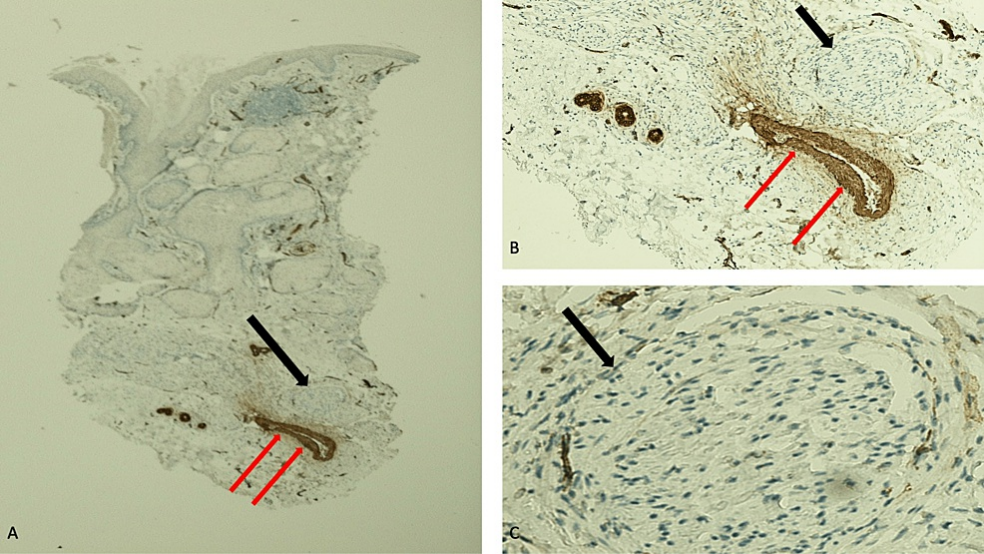
The nasal neuroma does not show staining with smooth muscle actin (SMA) stain Lower (A) and higher (B and C) magnification views of the pathology changes of the nasal neuroma do not show immunoexpression of SMA; the black arrow highlights the absence of SMA staining in one of the bundles of nerves in the reticular dermis. However, strongly positive SMA staining is observed in the smooth muscle-containing blood vessel wall (red arrows) [SMA immunoperoxidase stain: A, x2; B, x10; C, x40].

Immunoperoxidase staining was performed to confirm the identity of the spindle-shaped cells. The solubility of proteins in 100 percent ammonium sulfate (S100) stain showed strongly positive diffuse staining that highlighted the cells of the nerve bundles in the reticular dermis (Figure 4). The cluster of differentiation 34 (CD34) stain not only showed moderately positive diffuse staining of the neural tumor but also faint to weakly positive diffuse staining of the dermal stroma; in addition, the endothelial cells of large vessels in the deeper dermis and normal components of the epithelium in the upper dermis demonstrated positive staining with CD34 stain (Figure 5). The smooth muscle actin (SMA) stain did not show any positivity for the neoplasm in the deeper dermis; however, the blood vessel wall, which contained smooth muscle, demonstrated strongly positive staining (Figure 6).

Correlation of the patient’s clinical history, lesion morphology, and pathologic findings established a diagnosis of a traumatic neuroma with an overlying dilated pore. Most of the benign dermal neural neoplasm had been removed during the biopsy. However, residual tumor extended to the lateral margins of the specimen.

The patient continued to experience pain in her nasal tip after the biopsy site had healed. A trial of intralesional triamcinolone acetonide was suggested; however, she refused to have any injections into her nose. Her nose pain had occurred while she was using gabapentin; therefore, treatment with pregabalin was not recommended. Oral carbamazepine was suggested; she appreciated the recommendation and decided to follow up with the doctor who managed her fibromyalgia and back pain for alternative non-surgical interventions for her nasal tenderness.

## Discussion

A traumatic neuroma is typically associated with either a prior amputation (and has been referred to as a terminal neuroma) or an acute injury to the peripheral nerve [4-9]. However, a traumatic neuroma can develop from chronic and repetitive friction to a non-dissected nerve [10,16]. These have been referred to as either a spindle neuroma or a neuroma-in-continuity [5,6,9,10].

Most traumatic neuromas are asymptomatic and occur post-amputation on the lower extremity [4-6,11]. However, when symptoms occur, the tumor can drastically alter the quality of the patient’s life [4,8,9]. In addition to pain, a traumatic neuroma can produce cold intolerance, electrical sensitivity, numbness, and/or paresthesia [5,7,9,11].

The nose is an extraordinarily rare location for a traumatic neuroma. It is remarkable, considering the large number of elective cosmetic procedures performed on the nose, that only three individuals with a symptomatic traumatic neuroma at this site have been described. The characteristics of the patients with a traumatic neuroma of the nose are summarized in Table 1 [14,15].

**Table 1:** Characteristics of patients with a traumatic neuroma of the nose DM: Diabetes mellitus; FM: Fibromyalgia; HLD: Hyperlipidemia; HTN: Hypertension, HypoT: Hypothyroidism; LBP: Low back pain. ^a^The patient developed nasal pain, that was most prominent, at the columella and membranous septum four weeks after her rhinoplasty. The pain continued to increase over time. The pain was intolerable on evaluation one year after the operation. ^b^The patient lacerated his lip (immediately below the columella) at age four years; it was sutured. At age 11 years, a firm painless mass developed on the left side of the columella after blunt trauma to the previously injured area of his lip. From age 14 years, the mass progressively increased in size. He was evaluated at age 16 years. ^c^There was no penetrating trauma or surgery. ^d^At the caudal end of the septal cartilage, the investigators made a through-and-through (transfixion) incision and discovered the neural tissue. ^e^The surgical site (columella) had healed within two weeks after the biopsy; both sides of the nose were symmetrical. Four weeks later, the patient’s nasal columella was hit by a football. By 10 weeks post-biopsy, a 1.0 x 1.5-centimeter firm reddish mass had appeared on the left side of the columella; an incisional biopsy showed not only chronic inflammation with focal foreign body reaction but also fibrosis. There was no neuroma. Injection of intralesional triamcinolone acetonide was performed twice: 10 days after surgery (four millimeters of a 3.3 milligram per milliliter solution) and one month later (0.25 milligrams). The area healed without evidence of the mass or discoloration; the columella was symmetric.

Features	Patient one	Patient two	Patient three
Age	16-years-old	Not stated	64-years-old
Race	Caucasian	Turkish	Caucasian
Gender	Man	Woman	Woman
Medical history	None	None	DM, FM, HLD, HTN, HypoT, and LBP
Onset age	Four years	Four weeks postoperatively^a^	61 years
Symptom onset age	11 years	Four weeks postoperatively^a^	61 years
Duration	12 years	52 weeks	Three years
Precipitating event(s)	Left upper lip laceration, blunt trauma^b^	Aesthetic rhinoplasty	Recurrent insect bites, inflamed hair follicles, and rosacea flares^c^
Nasal location	Left medial crus	Columella and membranous septum	Nasal tip
Symptoms	Painless enlarging mass	Pain	Pain
Morphology	Firm nodule	Normal appearing nose	Bulbous nasal tip and dilated pore with a central depression
Size	1.0 x 1.5-centimeter nodule	Neuromatous structures^d^	Four x four millimeter umbilicated papule
Pathology diagnosis	Neuroma (traumatic)	Neuroma (traumatic)	Neuroma (traumatic) with overlying dilated pore
Treatment	Excisional biopsy	None described	Incomplete removal by punch biopsy; no additional treatment
Follow-up	Complete resolution^e^	None described	Pain persisted
Reference	[14]	[15]	Current report

The patients with a nasal traumatic neuroma included two women and one man. One of the women was Turkish, and her age at neuroma diagnosis was not stated. The other woman was Caucasian, and she was 64 years old when her neuroma was diagnosed. The man was also Caucasian; his neuroma was diagnosed at age 16 years [14,15].

The Caucasian woman has multiple medical problems. These included diabetes mellitus, fibromyalgia, hyperlipidemia, hypertension, hypothyroidism, and low back pain. The other patients did not have any comorbidities [14,15].

The onset age of the neuroma varied. The man was four years old when he lacerated his left upper lip, and it was sutured. Neuroma symptoms (consisting of a painless mass on the left medial crus of his nose) began when he experienced blunt trauma to the same lip site at age 11 years. The mass progressively enlarged, beginning at age 14 years; he was 16 years old when he was evaluated [14].

The Turkish woman had an aesthetic rhinoplasty; within four weeks after the surgery, she developed pain localized to the columella and membranous septum of her nose. The pain not only persisted but also continued to increase postoperatively. One year after her operation, the pain had become intolerable [15].

The Caucasian woman’s symptoms began when she was 61 years old. She had not experienced a penetrating injury or surgical operation on her nasal tip; however, the location was the site of recurrent insect bites, inflamed hair follicles, and rosacea flares. She had several evaluations since the onset of her unresolved nasal pain; the diagnosis of neuroma was finally established when she was 64 years old.

The duration of the nasal neuroma ranged from one year to 12 years (median, three years) before confirming the diagnosis. Both women had severe and unabating pain localized to the site of the tumor. The man’s lesion was painless [14,15].

The morphology of the benign neoplasm varied. The Turkish woman had a normal-appearing nose that contained neuromatous tissue that was discovered after a transfixion incision at the caudal end of the septal cartilage of her nose was performed. The other woman had a four-by-four millimeters umbilicated papule on the erythematous and bulbous tip of her nose. The man had a firm 1.0 by 1.5-centimeter nodule on the left medial crus of his nose [14,15].

A traumatic neuroma is a benign non-encapsulated neoplasm; a microscopic examination of the lesion shows a disorganized and tangled tumor composed of bundles that contain axons, endoneurial cells, nerve fibers, and perineurial cells surrounded by fibroblasts, collagenous connective tissue, and/or scar [4-6,10]. The three patients with a traumatic nasal neuroma had either a prior history of penetrating trauma or the observation of the removed tissue demonstrating an irregular arrangement of the nerve fibers diagnostic of a traumatic neuroma or both [14,15]. In addition, the Caucasian woman had a dilated pore in the upper dermis, overlying her traumatic neuroma.

Immunoperoxidase studies were only performed for Caucasian women. Similar to another traumatic neuroma, diffuse, strongly positive staining of the tumor cells was demonstrated with S100 stain [16-18]. In addition, diffuse--yet less dense--moderately positive CD34 staining was observed; it has been suggested that some of the positive staining cells represent endoneurial fibroblasts within the tumor [17-19]. In addition, faint to weakly positive diffuse CD34 staining of the dermal stroma was observed. The tumor cells of neuromas do not show positive staining for SMA; although one group of investigators observed that patients with a painful traumatic neuroma demonstrated diffusely distributed positive SMA staining in the cytoplasm of the proliferative myofibroblasts among the regenerated nerve fibers, neither the traumatic neuroma nor its associated myofibroblasts from the woman in this report had SMA immunoexpression [20]. 

The neuroma was completely removed during the excisional biopsy of the man’s tumor. Additional therapy, following the incomplete removal of the Caucasian woman’s neoplasm, was declined by the patient. Neuroma treatment was not described for Turkish woman [14,15].

The biopsy site on the nasal tip of the Caucasian woman healed without any complications. However, after the biopsy of her neuroma, she continued to have persistent pain. The follow-up for the Turkish woman was not described [14,15].

The man experienced blunt trauma to the excision site of his neuroma, and a similar-sized asymptomatic nodule developed. An incisional biopsy showed fibrosis accompanied by chronic inflammation and a foreign body reaction. The site completely healed--with a symmetric columella and without any residual mass or discoloration--following two monthly injections of triamcinolone acetonide [14].

The pathophysiologic mechanism for the development of pain associated with a neuroma remains to be established. Several hypotheses--which are not necessarily mutually exclusive--have been postulated. These include not only peripheral and central sensitization of the affected nerve but also the involvement of alpha-smooth muscle actin, nerve growth factor, and/or structural changes in neural fibers [4,7].

The management of a painful neuroma includes prevention, non-surgical treatment, and surgical intervention [4-13]. Prevention of neuroma may be executed during planned limb amputations. The resected nerve is cut proximally, and the free end may spontaneously reposition in a site that avoids not only the scar or damaged tissue but also mechanical stimulation from compression or friction. Alternatively, the distal nerve is buried in a muscle, bone, or vein [4-6].

Non-surgical treatments include systemic or local pharmacologic agents, local chemical interventions, cryotherapy, and physiotherapy. Oral drug therapies include alpha-receptor agonists, carbamazepine (a sodium channel blocker), pregabalin (a voltage-dependent calcium channel blocker), and opioids. Injectable intralesional agents that have been used are anesthetics, corticosteroids, etanercept, or onabotulinum toxin A [4,5,7,8].

Topical medications have included analgesics (such as ziconotide which can have neurologic-related adverse events, including abnormal gait, ataxia, dizziness, nausea, and nystagmus) and anesthetics (such as lidocaine). In addition to topical regional application, other routes of drug delivery for the management of a traumatic neuroma include intrathecal injection and intravenous infusion. Another treatment for a traumatic neuroma that is not specifically localized to the neuroma involves electrode-implantation into the spinal cord and subsequent pulse current stimulation [4,7]. 

Chemical interventions for a traumatic neuroma include not only intralesional corticosteroids but also ablation with neurolytic agents such as alcohol and phenol; however, leakage and local tissue necrosis are potential adverse events associated with ablation [4,7]. Decreased pain sensitivity and long-lasting analgesia have resulted from cryotherapy; a residual limb neuroma was effectively treated with an ultrasound-guided application of low temperature [4]. Physiotherapy (including percussion, therapeutic massage, and ultrasound) and neuromodulation have also been used to treat a traumatic neuroma [5-7,12].

There are several potential surgical interventions for a traumatic neuroma. Direct repair at the time of injury--before the development of the neuroma--is optimal. Otherwise, the simplest surgical method for treating a traumatic neuroma is ligation of the nerve ending; after proximal ligation, the nerve can remain in a site that will minimize subsequent irritation. Alternatively, there are many options for the management of the distal free end of the proximal nerve: burying it in muscle or bone, attaching it to a vein, capping the nerve end, reconstruction of the nerve continuity, or covering the nerve with a local skin flap [4,6-9,13].

An acronym is a mnemonic device that is used as a memory aid. Explosives--such as 2,4,6-trinitrotoluene (which also has the acronym TNT)--are used for industrial, military, and mining applications. The dramatic (and occasionally explosive) manifestation of tumor-associated pain from our patient’s traumatic neuroma of the nose prompted us to introduce the acronym for this lesion: TNT neuroma.

## Conclusions

A traumatic neuroma may be associated with severe pain. It typically occurs at the site of a prior amputation of the lower extremity. However, albeit rarely, a traumatic neuroma has been described on the nose. A Caucasian woman with an extremely painful nasal traumatic neuroma on her nasal tip is reported; the other two patients with this tumor on their nose include a Turkish woman and a Caucasian man. The traumatic neuroma of the nose was extraordinarily tender (in both women) and occurred following penetrating injury to the skin (in the Turkish woman and the man). Evaluation of the tissue specimen obtained after a biopsy of the affected area established the diagnosis. Follow-up was provided for two of the patients; the man never developed tumor-associated tenderness and his neuroma had been entirely removed, whereas even after the biopsy healed, excruciating pain persisted for the reported woman whose neuroma had not been completely excised. Prompted by the reported patient’s explosive and markedly severe lesion-associated pain, we proposed the acronym TNT neuroma to emphasize the symptom (tender), the location (nasal), and the variant (traumatic) of this rare type of neuroma.
